# Tunable terahertz fishnet metamaterials based on thin nematic liquid crystal layers for fast switching

**DOI:** 10.1038/srep13137

**Published:** 2015-08-14

**Authors:** Dimitrios C. Zografopoulos, Romeo Beccherelli

**Affiliations:** 1 Consiglio Nazionale delle Ricerche, Istituto per la Microelettronica e Microsistemi (CNR-IMM), Roma 00133, Italy

## Abstract

The electrically tunable properties of liquid-crystal fishnet metamaterials are theoretically investigated in the terahertz spectrum. A nematic liquid crystal layer is introduced between two fishnet metallic structures, forming a voltage-controlled metamaterial cavity. Tuning of the nematic molecular orientation is shown to shift the magnetic resonance frequency of the metamaterial and its overall electromagnetic response. A shift higher than 150 GHz is predicted for common dielectric and liquid crystalline materials used in terahertz technology and for low applied voltage values. Owing to the few micron-thick liquid crystal cell, the response speed of the tunable metamaterial is calculated as orders of magnitude faster than in demonstrated liquid-crystal based non-resonant terahertz components. Such tunable metamaterial elements are proposed for the advanced control of electromagnetic wave propagation in terahertz applications.

Metamaterials (MM) are artificial structures characterized by features on a sub-wavelength scale, which exhibit fascinating properties not found in their natural counterparts. By proper design, for instance, one can engineer their permittivity and permeability values, which can lead to negative refractive index and associated physical phenomena, e.g. negative refraction, antiparallel phase velocity, or subwavelength focusing^1^. Owing to such exotic possibilites, they are envisaged as the core element in a broad range of applications spanning from beam steering and lenses^2^, electromagnetic (EM) wave modulators and absorbers^3^, to chemical and bio-sensors^4^ or electromagnetic cloaking techniques^5^,^6^. Since the first experimental demonstrations of negative index metamaterials (NIM) in the microwave spectrum^7^,^8^, various NIM structures have been investigated from microwave up to optical frequencies, among which the fishnet structure, whose typical configuration consists of a dielectric slab cavity formed between two identical periodic arrays of interconnected metallic patches. Fishnet metamaterials provide ease of fabrication and scalability of their properties in a wide spectral range^9^,^10^, which has been demonstrated from the microwave to the near-infrared (NIR) and visible spectrum^11^,^12^.

In particular, in the frequency interval between microwaves and infrared waves, the so-called THz gap, fishnet metamaterials have been under intense investigation in view of novel THz wave manipulation devices^13^,^14^. These are expected to boost the rapidly advancing field of THz technology, and its numerous envisaged applications, such as chemical detection of hazardous materials, safe bio-imaging and detection of diseases, inherently secure short-range communications, and non-destructive testing in industry^15^. However, fully exploiting the potential of metamaterials as functional components in THz science would also demand for a means of dynamically tuning their properties, a key aspect in modulating, switching, steering, and filtering devices.

In this context, nematic liquid crystalline (LC) materials offer a promising solution, as they have been long used as the active element in electric or optical field-actuated photonic devices^16^,^17^,^18^,^19^, including as well fishnet metamaterials^20^. Owing to their large inherent anisotropy and capacitive operation, LC-based tunable devices feature very low power consumption^21^, combined with large refractive index modulation and polarization control features. These properties characterize nematic materials not only in the visible or NIR spectrum, but can be extended down to terahertz and microwave frequencies^22^,^23^, thus enabling the design of functional components, such as varactors^24^, phase shifters and modulators^25^,^26^, beam steerers^27^,^28^, reflectarrays^29^,^30^,^31^, absorbers^32^,^33^,^34^, and tunable metamaterials or frequency selective surfaces^35^,^36^,^37^. In the THz spectrum, the infiltration of metamaterial resonant structures with nematic materials offers a striking advantage, namely the reduction of the LC layer thickness to few microns, owing to their large interaction with the resonant EM field^26^,^34^,^36^,^37^. Such dimensions are compatible with standard LC technology used in the photonics or display industry, they eliminate alignment issues that may appear in thicker non-resonant cells thus far employed in THz phase modulators and filters^38^,^39^, require low driving voltages, and, most importantly, allow for orders of magnitude faster switching speeds, since the response times of nematic LC cells scale with the square of their thickness.

Here, we investigate a class of LC-tunable fishnet metamaterials, designed to work in the proximity of 1 THz. Apart from providing the electromagnetic resonances that lead to NIM properties, the metallic layers also serve as the electrodes for the application of the control voltage. The latter allows for the tuning of the metamaterial key parameters, such as transmittance and effective permittivity or permeability. Both the LC switching characteristics and the EM properties of the LC-THz-MM are rigorously investigated by means of finite-element based numerical tools. It is demonstrated that via the switching of a nematic mixture with high anisotropy at THz, the magnetic resonance of the MM can be tuned in a range of 150 GHz. Subsequently, the EM properties of the MM can also be controlled via the applied voltage, e.g. the effective refractive index for normal incidence, which can be tuned from positive to negative values. The device is engineered so that the dynamics of the metamaterial’s EM response are faster than that of the LC switching. Switching times nearly compatible with video rate operation are predicted, namely orders of magnitude lower than those in other LC-tunable THz devices^39^,^40^,^41^.

The paper is organized as follows: after the introduction, Section II investigates into the EM properties of a class of fishnet THz metamaterials and their maximum achievable tuning range by employing a high-Δ*n* nematic mixture optimized for THz LC-based applications. Section III presents a rigorous study of the electrically controlled LC-tunable properties for a particular design that allows for transmission modulation and tuning of the effective MM index. Finally, conclusions are drawn in Section IV.

## Liquid-crystal tunable fishnet metamaterials

**Figure 1:**
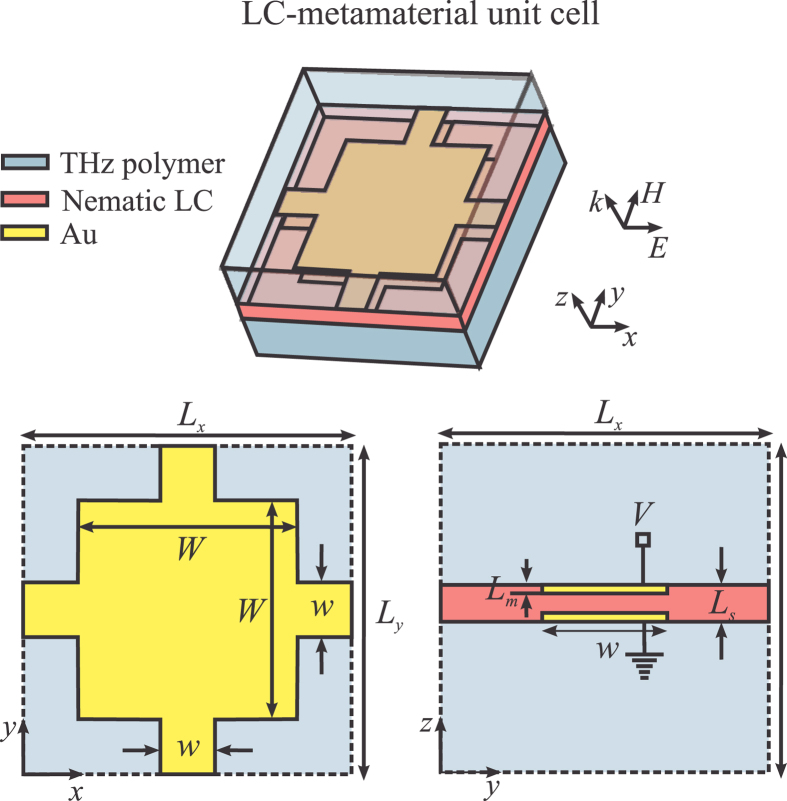
Schematic layout and geometrical parameter definition of the proposed LC-tunable fishnet terahertz metamaterial. The fishnet metallic network is patterned on a low-loss THz dielectric substrate and a liquid-crystal cavity is formed between two opposing fishnet slabs.

The layout of the proposed LC-THz-MM is shown in Fig. 1. The fishnet periodic structure is made of gold and is characterized by the lattice constant *L*_*x*_ = *L*_*y*_ = 150 μm, the square patch dimension *W*, the stripe width *w*, and it is patterned on an infinite substrate of a low-loss THz polymer, e.g. Zeonor with a refractive index *n*_*p*_ = 1.518^42^. The metal thickness layer is *L*_*m*_ = 300 nm and that of the LC-dielectric layer *L*_*s*_ = 10 μm. The thickness *L*_*s*_ is controlled by placing dielectric spacers in the edges of the device, away from its active central region, so that they do not influence its EM properties. The permittivity of gold is described by the Drude model


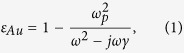


where *ω*_*p*_ = 1.37 × 10^16^ s^−1^ is the plasma and *γ* = 4.05 × 10^13^ s^−1^ is the scattering frequency^10^.

The LC material is the high-Δ*n* mixture 1825 characterized by THz complex ordinary and extraordinary refractive indices equal to *n*_*o*_ = 1.554 − *j*0.018 and *n*_*e*_ = 1.941 − *j*0.022 (1 THz), low-frequency (1.5 KHz) permittivities *ε*_*o*_ = 4.7 and *ε*_*e*_ = 21.7, elastic constants *K*_11_ = 12.5 pN, *K*_22_ = 7.4 pN, *K*_33_ = 32.1 pN, and rotational viscosity *γ*_1_ = 311.55 mPa·s^43^,^44^. This particular nematic mixture has been selected as it exhibits the highest thus far demonstrated Δ*n* at THz frequencies, low dichroism, and moderate losses. When no voltage is applied, the LC molecules show a homogeneous alignment along the *x*−axis, promoted by a thin alignment layer. This layer is typically formed by rubbed polymers, e.g. polyimides, or photoalignment materials. In the latter case, it is a monomolecular layer of few nanometers and thus does not influence the EM properties of the device. In the case of rubbed polymers, the layer’s thickness is a few tens of nanometers, i.e. deeply subwavelength with respect to the impinging THz wave, and almost index-matched to the substrate. Therefore, it has not been included in the analysis in order to avoid unnecessary conditioning of the finite-element mesh. The voltage is applied between the two fishnet films in order to induce the reorientation of the LC molecules, and therefore of the anisotropy axis, along the *z* direction. The local molecular orientation is described via a unit vector **n**, termed as the director. We define two limiting cases, namely when the LC molecules are unbiased (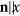) and when they are fully switched (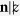), which determine the maximum tunability provided by the LC material.

**Figure 2:**
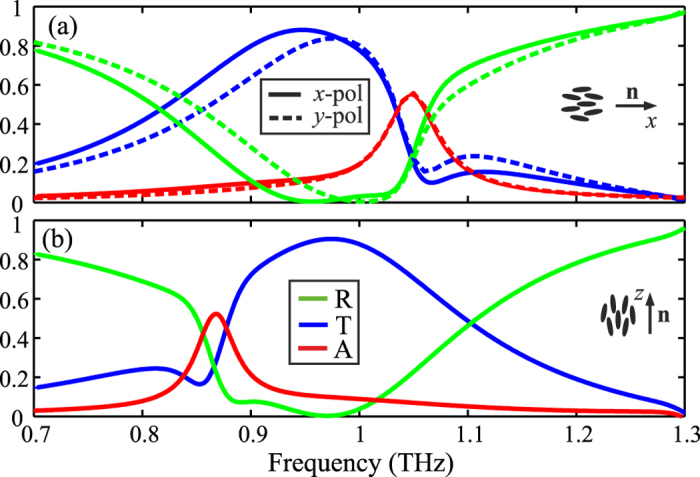
Reflectance, transmittance, and absorption spectra of the investigated LC-tunable THz fishnet metamaterial characterized by *W* = 110 μm and *w* = 40 μm, for the limiting LC alignment configurations, namely along along the (**a**) *x*− and (**b**) *z*−axis. The polarization dependence of the metamaterial response is also studied in (**a**), indicating moderate polarization sensitivity for different LC molecular orientation in the *x*-*y* plane. No polarization dependence exists when **n** is parallel to the *z*-axis.

**Figure 3:**
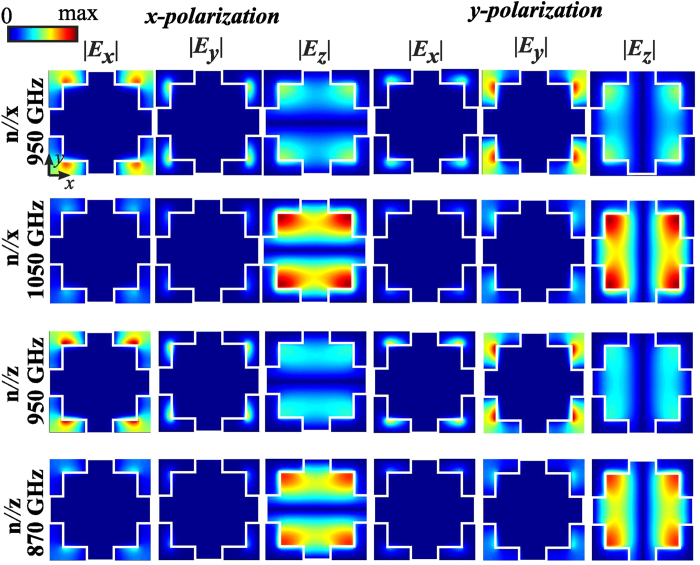
Spatial profile of the three components of the electric field for the non-resonant frequency *f*_0_ = 0.95 THz and the two resonant frequencies *f*_*H*_ = 1.05 THz and *f*_*L*_ = 0.87 THz, which correspond to the LC molecules aligned along the *x*− and *z*−axis, respectively. The fishnet dimensions are *W* = 110 μm and *w* = 40 μm.

Figure 2 shows the transmittance, reflectance, and absorption spectra of the fishnet LC-THz-MM, for *W* = 110 μm and *w* = 40 μm, calculated for both limiting cases and asssuming *x*− or *y*−polarization for a perpendicularly impinging plane wave. It is remarked that for the lattice constant here considered, the cutoff frequency for the lowest non-zero diffraction order in the polymer substrate is above the spectral window under investigation^34^. Calculations were performed by means of the finite element method^45^, imposing periodic boundary conditions at the side-walls of the unit cell shown in Fig. 1. A broadband transmission window centered at approximately 0.95 THz can be observed for both polarization and LC orientations. This corresponds to the EM mode supported through the swiss-cross shaped apertures formed in-between the fishnet metallic structures. The corresponding field profiles calculated at the *z* = 0 plane, shown in Fig. 3, verify that at 0.95 THz the electric field is mainly confined in the apertures, resembling a TE_10_ mode, which is excited since the length of the swiss-cross is adequate to provide high transmittance (cf. the limit of *L*_*y*_/2 in the case of a periodic array of subwavelength square holes^46^). Although the structure itself is symmetric, owing to the presence of the LC material, there is a degree of anisotropy in the EM response in the rest case, which is investigated in Fig. 2(a). On the contrary, in the fully switched case, the response is polarization-independent, as both polarizations of the plane wave sense the ordinary LC index.

Shifting within the high transmittance window, the absorption peaks observed in Fig. 2 indicate the presence of a resonant EM gap plasmon mode, which is the one responsible for the metamaterial effect. Close to the resonant frequency the EM field is confined almost exclusively between the metallic patches, as demonstrated in Fig. 3, and forms a current loop that induces a magnetic resonance^9^,^47^. The field profiles of Fig. 3, calculated at the resonant frequencies for the two LC alignment limit cases, demonstrate that the associated gap plasmon mode has an electric field with a predominant *E*_*z*_ component, and thus shows very small dependence on the polarization of the impinging wave, as evidenced in the absorption spectra of Fig. 2(a). The spectral position of this resonance depends on the refractive index of the dielectric slab material along the *z*−axis, and it can be efficiently tuned by controlling the LC molecular orientation. A shift of 180 GHz is predicted from the unbiased to the fully switched state, as also designated by the corresponding absorption peaks in Fig. 2(a,b), respectively.

**Figure 4:**
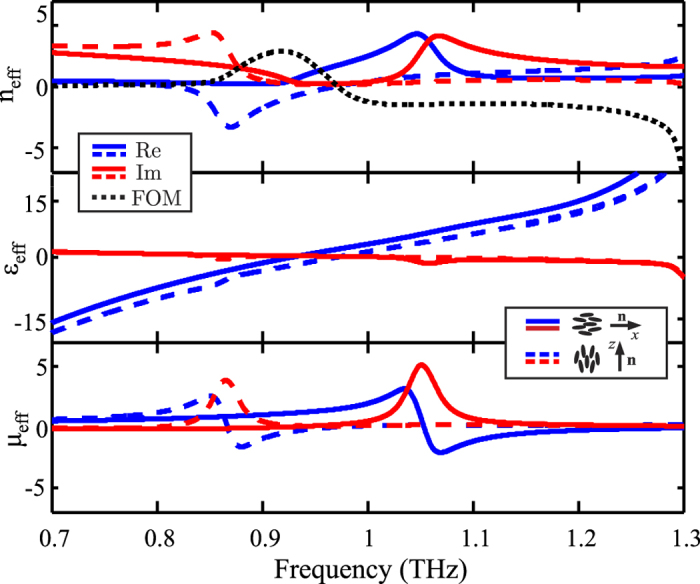
Effective parameters of the LC-THz metamaterial under study (*W* = 110 μm and *w* = 40 μm) for the two extreme cases of LC molecular orientation, i.e. along the *x−* (rest case) and the *z*−axis (fully switched alignment).

In parallel, the stripes connecting the metallic patches lead to an effective permittivity response of the metamaterial, whose cross-over frequency *f*_*co*_, i.e. where *ε*_eff_ = 0, can be adjusted by varying *w*. A negative MM index is possible, when both the permittivity and permeability values of the MM become negative. For the case of normal incidence, these are calculated in Fig. 4, following the procedure described in Ref. 48, and starting from the calculated transmission and reflection coefficients for a MM thickness *d*_MM_ = *L*_s_. The permeability spectra of Fig. 4(c) confirm the tuning of the magnetic resonance, and correspond to the same shift observed in the absorption peaks of Fig. 2. On the contrary, the effective MM permittivity does not depend significantly on the LC dielectric slab index (Fig. 4(b)), and thus by shifting the magnetic resonance from *f* > *f*_*co*_ to *f* < *f*_*co*_ the sign of the MM index *n*_MM_ is reversed, as shown in Fig. 4(a). The peak of the high transmission windows in Fig. 2 coincides with the frequency where *n*_MM_ ≃ *n*_*p*_, indicating impedance matching. In the fully switched case *n*_MM_ is negative in the interval 0.81 < *f* < 0.97 THz, while at 0.92 THz the figure of merit, defined as −Re{*n*_eff_}/Im{eff}, is maximized.

**Figure 5:**
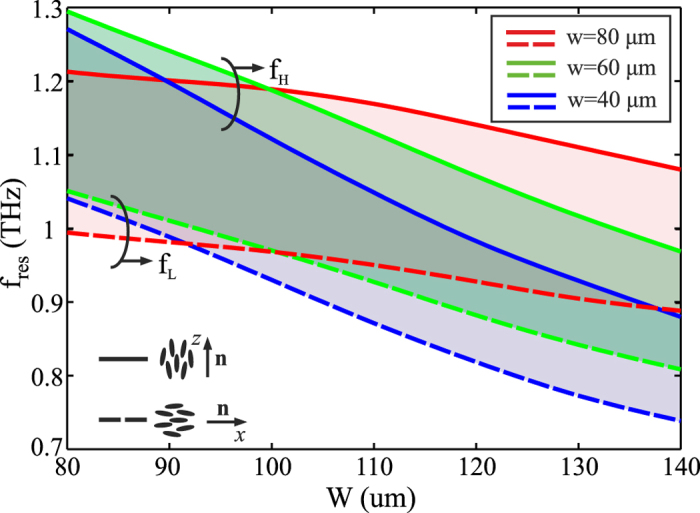
Resonant frequency of the fishnet metamaterial as a function of the patch size *W* and the interconnecting stripe width *w* for the two limiting cases of the LC alignment. The shaded areas indicate the available tunability range offered by the selected high-Δ*n* nematic compound 1825, showing that the resonance can be tuned by more than 200 GHz. The high/low resonant frequencies *f*_*H*_/*f*_*L*_ correspond to the unbiased and fully switched cased, respectively.

**Figure 6:**
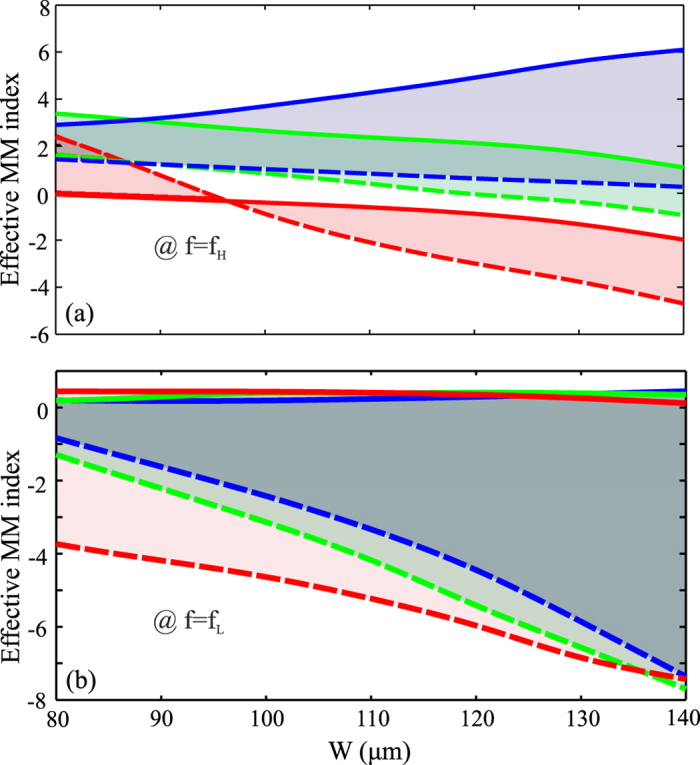
Effective metamaterial index calculated at the (**a**) high and (**b**) low resonant frequencies of the fishnet metamaterials for various geometrical parameters and for both limiting cases of the LC alignment (legend as in Fig. 5). The shaded areas indicate the available tunability range offered by the selected nematic material.

The main observations concluded for this specific design (*W* = 110 μm and *w* = 40 μm) are also valid when the MM structural parameters are varied. The resonant frequencies as a function of *W* and *w* for the investigated LC-THz-MM are shown in Fig. 5, for both limiting cases of the LC alignment. The shaded areas indicate the tunability range achievable by switching between the two LC states, showing that tuning ranges higher than 200 GHz are possible for the selected nematic material. The corresponding variations of the effective MM index are shown in Fig. 6, calculated at the two resonant frequencies *f*_*H*_ and *f*_*L*_, which correspond to the rest and fully switched cases, respectively. The MM index at *f*_*L*_ is slightly positive when the MM is off-resonance and obtains a large negative value on-resonance, revealing the possibility for continuous tuning from positive to zero and finally negative values. The corresponding behaviour at *f*_*H*_ is more complex, since the exact value of *w* determines the position of *f*_*co*_ and therefore the sign of the MM index.

## Dynamic control of liquid-crystal terahertz fishnet metamaterials

In the previous Section, we have examined the LC-THz-MM response for the two limiting LC alignment states, which determine the maximum achievable tuning range. Here, we investigate into the voltage-dependent tunable properties of the fishnet LC-THz-MM and their dynamics by rigorously solving for the LC addressing problem. This is performed by employing the *Q*−tensor formulation, an advanced numerical tool for the accurate studies of the LC orientation in confined geometries. The employed model is capable of capturing edge effects in all three dimensions, defect singularities, and nematic order parameter variations, thus further advancing state-of-the-art approaches in LC modeling in microwave or terahertz components^49^. In the most general case of biaxial nematic configurations the traceless and symmetric **Q** matrix can be expressed as


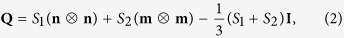


where **I** is the unitary matrix and **n**, **m**, and **n** × **m** are its eigenvectors with corresponding eigenvalues (2*S*_1_ − *S*_2_)/3, (2*S*_2_ − *S*_1_)/3, and −(*S*_1_ + *S*_2_)/3. Purely uniaxial solutions exist when two eigenvalues are equal leading to **Q** = *S*[(**n**⊗**n**) − (1/3)**I**], where *S* = *S*(*x*, *y*, *z*) is the nematic order parameter.

The total energy in the LC bulk is composed by three contributions expressed via the thermotropic, elastic, and electromagnetic energy density functions. Following the notation of Ref. 50, the thermotropic coefficients are equal to *a* = −0.3 × 10^5^ J/m^3^, *b* = −1.5 × 10^5^ J/m^3^, and *c* = 2.5 × 10^5^ J/m^3^ 51, leading to an equilibrium order parameter equal to *S*_eq_ = 0.6, a value usually found experimentally for liquid crystals in the nematic state. The elastic coefficients used in the model are 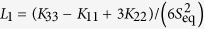, 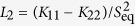, and 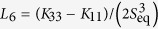, where *K*_*ii*_ are the corresponding Frank elastic constants. The electrostatic energy in the presence of the low-frequency control electric field, which is applied using the metallic fishnet layers as electrodes, is 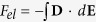, where 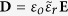 is the displacement field. The relative permittivity tensor is given by 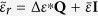, 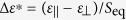 being the scaled dielectric anisotropy and 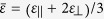. Hard anchoring conditions along the *x*−axis are applied at the top and bottom boundaries of the LC cell, with a pretilt angle of 2°.

The total free energy of the system is minimized by solving the Euler-Lagrange equations given by


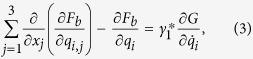


for *i* = 1…5, where *q*_*i*,*j*_ = ∂*q*_*i*_/∂*x*_*j*_, *x*_*j*_ being the unit vectors of the three-dimensional cartesian system, and *F*_*b*_ the total energy density function, composed by the thermotropic, elastic, and electrostatic contributions, as described in detail in Ref. 50. The r.h.s. of (3) describes the dynamic evolution of the **Q** tensor via the dissipation function 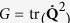, where 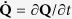, thus allowing for the study of the switching dynamics of the proposed device. The term 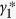 is related to the LC rotational viscosity *γ*_1_ via 
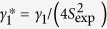
, where *S*_exp_ = *S*_eq_ is the order parameter of the LC during the measurement of its physical properties^52^. When a field is applied across the LC cell, the set of equations (3) is coupled with Gauss’ law ∇ ⋅ **D** = 0, in order to calculate the spatial variation of both the LC orientation profile via *q*_*i*_(*x*, *y*, *z*) and the electric field potential *V*(*x*, *y*, *z*).

**Figure 7:**
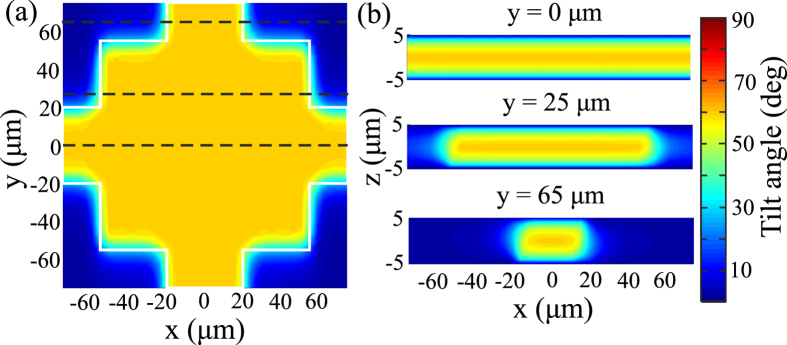
Tilt angle profile. (**a**) in the *x*-*y* plane and (**b**) in three *x*-*z* planes indicated by dashed lines in (**a**), for an applied voltage of 2 V. The liquid-crystal molecules switch in the volume between the metallic fishnet electrodes, with some fringe-field effects near their borders. The tilt angle profile across the main volume of the metamaterial is typical of Fréedericksz transition in nematic liquid-crystal cells.

The application of the low-frequency control voltage with a root-mean-square value *V* between the two fishnet electrodes leads to the reorientation of the LC molecules. In the area between the metallic patches and stripes, the electric field points perpendicularly and induces a torque to the LC molecules. When the electric field intensity surpasses a certain threshold, the LC molecules tilt in the *x*-*z* plane, tending to align with the applied field. This LC switching behaviour resembles strongly the Fréedericksz transition in LC cells, as evidenced in Fig. 7, which plots the LC tilt angle profiles for *V* = 2 V, where the tilt angle is defined as the angle of the nematic director with respect to the *x*-*y* plane. The MM structural parameters are chosen as in the case investigated in Fig. 2. It is observed that between the electrodes the tilt angle is almost constant at the *x*-*y* plane, apart from some edge effects at the borders of the fishnet metallic structure. In the region away from the metallic network, the driving field intensity is not sufficient to induce a reorientation of the LC molecules. Nevertheless, it is important to point out that the magnetic resonance shift, which is the key aspect of the investigated LC-THz-MM, depends on the LC refractive index only between the electrodes, since this is where the gap plasmon mode is confined. In the *x*-*z* planes the tilt angle assumes a typical profile across the LC cell between the electrodes, as shown in Fig. 7(b), that obtains the maximum value at the mid-plane of the LC cell and a fixed value equal to the pretilt at the LC-metal interfaces, owing to the hard anchoring conditions.

**Figure 8:**
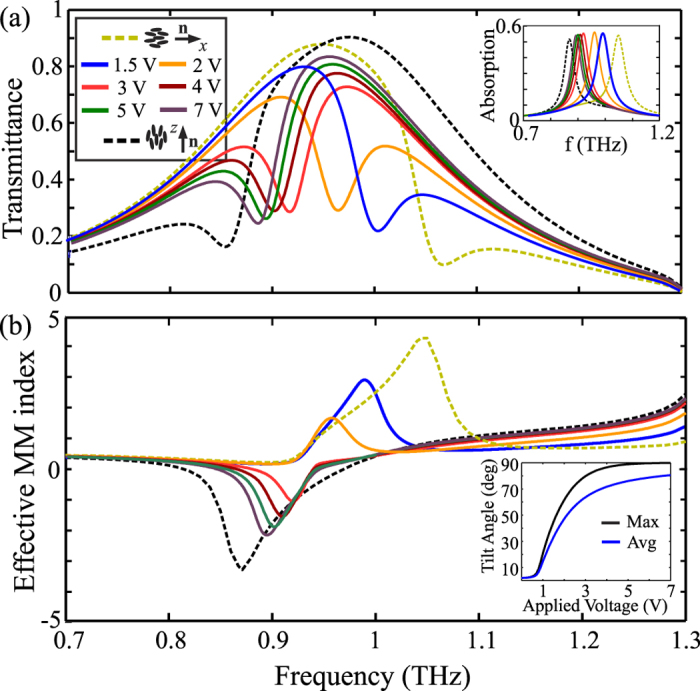
(**a**) Transmittance of the LC-THz metamaterial for various values of applied voltage. The limiting cases of resting and fully switched LC molecules are shown in dashed lines. The inset shows the corresponding absorption spectra, indicating a shift of the metamaterial resonance for increasing voltage values. (**b**) Effective metamaterial index for the cases studied in (**a**). In the range approximately between 0.9 and 1 THz, tuning from positive to zero to negative values is possible. The inset shows the maximum and average LC tilt angle. Owing to the hard anchoring conditions at the metallic surfaces, the resonance tuning effect saturates before the maximum tuning range is achieved.

The tuning of the metamaterial’s properties as a function of the applied voltage is investigated in Fig. 8. As the voltage is increased, the LC molecules are further tilted, the LC index along the *z*−axis moves from *n*_*o*_ towards the higher extraordinary value *n*_*e*_ and induces a progressive shift of the magnetic resonance towards lower frequencies, as demonstrated in the inset of Fig. 8(a). The EM response to the applied voltage is non-linear and the tuning efficiency saturates as higher voltages are applied. This is a typical characteristic of LC-tunable devices, owing mainly to the strong boundary conditions, which force the LC molecules to stay anchored at the cell’s surfaces, thus hindering the overall average switching of the LC molecules. This can be observed in the inset of Fig. 8(b), where the maximum and average LC tilt angle are calculated in the *x*-*y* plane at *z* = 0 and −*L*_*s*_/2 ≤ *z* ≤ *L*_*s*_/2, respectively. Nevertheless, Fig. 8 demonstrates that a moderate voltage of 7 V is sufficient to cover more than 80% of the full tunability range corresponding to the two limit cases, which translates in a shift of more than 150 GHz. Therefore, extensive tunability is achieved for rather low applied voltage values, a fact that is very important in terms of switching power consumption. The latter can be approximated as *P* = *CV*^2^*f*, where *f* is the LC driving frequency, and *C* the capacitance of the LC cell. Assuming a total MM area of 3 × 3 mm^2^ (20 × 20 unit cells) and for the fully switched case, the capacitance is below 1 nF and the overall power consumption obtains very low values, below 100 μW.

Based on the calculated spectra as in Fig. 8(a), the voltage-dependent tuning of the MM effective index is calculated in Fig. 8(b). A transition from positive to negative values of *n*_MM_ at the shifting resonant frequency is achieved by increasing the applied voltage. At a given frequency, the index can be tuned by properly adjusting the control voltage, as in the maximum tuning ranges investigated in Fig. 6. Therefore, the overall results of Fig. 8 demonstrate the capability of the proposed configuration to dynamically tune the EM properties of the fishnet THz metamaterial, via the addressing of an active LC-layer with a low voltage.

**Figure 9:**
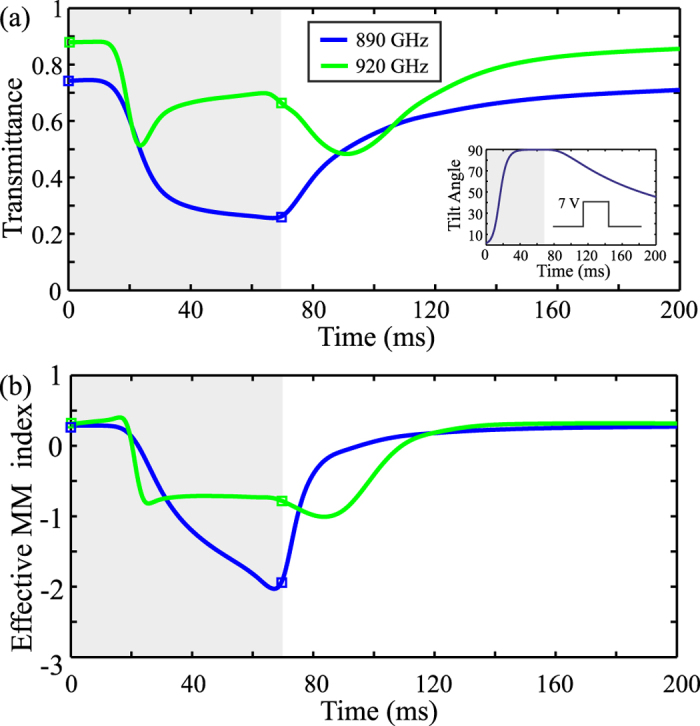
Transient study of the voltage-tunable metamaterial electromagnetic response for an applied rectangular voltage pulse of 7 V amplitude and 70 ms duration at two characteristic frequencies. 890 GHz (metamaterial resonance) and 920 GHz (maximum figure of merit). (**a**) Transmittance and (**b**) effective metamaterial index. The inset in (**a**) shows the dynamics of the tilt angle calculated at the center of the LC layer.

In order to study the metamaterial’s temporal response and dynamics, a rectangular voltage pulse of 7 V is applied between 0 and 70 ms. The inset in Fig. 9(a) shows the evolution of the maximum tilt angle, calculated at the center of the LC layer. The rise time depends on the applied voltage and is faster than the fall time, which is described by the physics of an exponential elastic relaxation^22^. However, the collective response of the metamaterial’s EM properties shows faster dynamics, as evidenced in Fig. 9, where the transmittance and the MM effective index are calculated as a function of time, for two characteristic frequencies: the magnetic resonance frequency (890 GHz) and the frequency at which the figure of merit is maximized (920 GHz). In the first case, both maximum transmittance modulation and refractive index change is achieved, while in the second the index modulation comes with a small change in transmittance. Also to be remarked, the transition of the MM index in the second scenario is not monotonic and both higher and lower values, compared to that of the steady-state, are observed during LC switching. This can be understood by noting the corresponding spectra in Fig. 8, indicating that the LC-tuning of the proposed THz-MM is governed by rich dynamics, which may provide an extra degree of freedom in the engineering of their properties. For instance, in the example investigated in Fig. 9 at *f* = 920 GHz, it is observed that the target value of the effective index at *t* = 70 ms can be achieved at approximately 20 ms with the same transmittance modulation. Thus, by overdriving the device at 7 V, the switch-on speed of the device can be significantly enhanced.

Owing to the few-μm thick LC cell employed, the proposed LC-THz-MM offers much faster response times than those in typical LC-based THz-components that, in order to achieve the desired phase or amplitude modulation, rely on thick LC layers (100 s of μm) with response times in the order or even much higher than one second^39^,^40^,^41^,^53^,^54^. Further pushing the response dynamics of such LC-THz-MM to lower values can be achieved by various means. In this work, we focused on the nematic mixture 1825, which offers the highest thus far reported THz anisotropy and consequently maximizes the tunability range. Other nematic mixtures optimized for THz applications are also available with significantly lower rotational viscosity values, e.g. less than 100 mPa/s^22^,^55^, which can reduce the response time by at least a factor of three, since the latter is proportional to *γ*_1_^22^. Moreover, optimizing the MM response for lower values of *L*_*s*_ can further improve the device switching speed. In addition, the use of dual-frequency LCs^56^ can eliminate the asymmetry between rise and decay times, by properly applying pulses at two different control frequencies so that the sign of the LC dielectric anisotropy Δ*ε* is inverted.

Finally, in applications where the maximum tuning range requirements are less strict, the use of moderate-Δ*n* fluorinated LC mixtures can significantly reduce the LC absorption losses at THz^57^, and thus the overall insertion losses of the device. In this work, the absorption losses in the LC bulk at the MM resonant frequencies were calculated as more than 65% of the total (the remaining owing to surface losses at the metal), indicating that the LC absorption is the dominant loss mechanism in the proposed THz fishnet metamaterials.

## Conclusions

In conclusion, the tunable properties of a THz fishnet metamaterial enhanced with a nematic LC layer have been theoretically investigated. The analysis demonstrates that the electrical addressing of the LC can shift the magnetic resonance by 150 GHz and tune the metamaterial properties. Voltage values below 10 V are sufficient to provide extensive tunability, thus implying sub-mW power requirements. The LC switching dynamics and the MM response have also been studied, predicting millisecond switching times, as in typical photonic LC-based components that employ μm-thick cells infiltrated with nematic materials.

## Additional Information

**How to cite this article**: Zografopoulos, D. C. and Beccherelli, R. Tunable terahertz fishnet metamaterials based on thin nematic liquid crystal layers for fast switching. *Sci. Rep.* **5**, 13137; doi: 10.1038/srep13137 (2015).
